# A Comparison of Behavioral Methods for Indexing the Auditory Processing of Temporal Fine Structure Cues

**DOI:** 10.1044/2019_JSLHR-H-18-0217

**Published:** 2019-05-30

**Authors:** Eric C. Hoover, Brianna N. Kinney, Karen L. Bell, Frederick J. Gallun, David A. Eddins

**Affiliations:** a Department of Communication Sciences and Disorders, University of South Florida, Tampa; b National Center for Rehabilitative Auditory Research, Portland VA Medical Center, Oregon; c Department of Otolaryngology–Head and Neck Surgery, Oregon Health and Science University, Portland

## Abstract

**Purpose:**

Growing evidence supports the inclusion of perceptual tests that quantify the processing of temporal fine structure (TFS) in clinical hearing assessment. Many tasks have been used to evaluate TFS in the laboratory that vary greatly in the stimuli used and whether the judgments require monaural or binaural comparisons of TFS. The purpose of this study was to compare laboratory measures of TFS for inclusion in a battery of suprathreshold auditory tests. A subset of available TFS tasks were selected on the basis of potential clinical utility and were evaluated using metrics that focus on characteristics important for clinical use.

**Method:**

TFS measures were implemented in replication of studies that demonstrated clinical utility. Monaural, diotic, and dichotic measures were evaluated in 11 young listeners with normal hearing. Measures included frequency modulation (FM) tasks, harmonic frequency shift detection, interaural phase difference (TFS–low frequency), interaural time difference (ITD), monaural gap duration discrimination, and tone detection in noise with and without a difference in interaural phase (N_0_S_0_, N_0_S_π_). Data were compared with published results and evaluated with metrics of consistency and efficiency.

**Results:**

Thresholds obtained were consistent with published data. There was no evidence of predictive relationships among the measures consistent with a homogenous group. The most stable tasks across repeated testing were TFS–low frequency, diotic and dichotic FM, and N_0_S_π_. Monaural and diotic FM had the lowest normalized variance and were the most efficient accounting for differences in total test duration, followed by ITD.

**Conclusions:**

Despite a long stimulus duration, FM tasks dominated comparisons of consistency and efficiency. Small differences separated the dichotic tasks FM, ITD, and N_0_S_π_. Future comparisons following procedural optimization of the tasks will evaluate clinical efficiency in populations with impairment.

There is an increasing awareness of patients in clinical settings who present with an audiogram that is within or near normal limits but who have extensive complaints regarding their difficulty with speech communication in the presence of background competition. Recent studies report some degradation in performance on measures of temporal fine structure (TFS) processing in aging populations, sensorineural hearing loss, and a history of traumatic brain injury (e.g., Füllgrabe, Moore, & Stone, 2015; Gallun, Diedesch, Kampel, & Jakien, 2013; Grose & Mamo, 2012; Hoover, Souza, & Gallun, 2017; Moore, 2008). Individuals in these populations frequently present with complaints of speech perception difficulty, especially in noisy environments, that is worse than predicted by their pure-tone audiogram. Although the audiogram is the most widely used clinical tool for the evaluation of hearing, the audiogram alone does not provide enough information to explain variation in speech perception abilities among patients and laboratory subjects. TFS processing ability is among a host of basic auditory abilities with potential clinical utility.

Clinical assessment of TFS has been suggested to expand our ability to detect and diagnose auditory disorders and to guide treatment decisions. There is preliminary evidence that TFS testing could provide objective validation of self-reported difficulty following brain injury (Gallun, Papesh, & Lewis, 2017) and may prove useful in detecting hidden hearing loss or distinguishing it from other cochlear pathologies (Paul, Bruce, & Roberts, 2017; Ridley, Kopun, Neely, Gorga, & Rasetshwane, 2018). TFS thresholds have been found to be predictive of self-reported benefit from hearing aid amplification in older adults with hearing loss (Perez, McCormack, & Edmonds, 2014), although this was not replicated in a recent study that included different predictive factors (Lopez-Poveda et al., 2017). However, in that study, Lopez-Poveda et al. (2017) found that TFS was predictive of aided speech intelligibility in older adults with hearing loss. These studies suggest that TFS testing has the potential to provide a useful metric for guiding clinical treatment decisions, including establishing hearing aid candidacy and predicting benefit.

Although there has been substantial progress in our understanding of how complex sounds are encoded and processed in the auditory periphery, brainstem, and cortex, there has not been a concurrent improvement in the tools available to clinicians. Tests recommended for the assessment of auditory processing are difficult to interpret in the context of current models of healthy and impaired auditory function (Cacace & McFarland, 2013). Until recently, many clinics were limited to presenting tests via audio CD and audiometer, and few clinics had the capability to set up, calibrate, and administer tests on a personal computer. As a result, the translation of laboratory tests into practical clinical tools required altering the psychometric properties of the test procedures for compatibility with clinical hardware requirements. Few studies have directly compared the differences between tests validated in laboratory studies using established psychophysical methods and the clinical versions of the same test administered using different methods adapted for clinical use. In a recent study, we found that gap detection thresholds measured using laboratory procedures accounted for less than half of the variance (*r* ^2^ = .49) scores on a popular clinical test of gap detection administered to listeners with a range of age and pure-tone thresholds (Hoover, Pasquesi, & Souza, 2015). The need to design a test around the tools available in a typical audiology clinic resulted in compromises in the methods that may have introduced confounding factors.

Recent advances in the availability of low-cost consumer devices capable of reproducing auditory tests within the tolerances of a laboratory experiment result in the ability to translate many laboratory measures into clinical tools without altering the psychometric properties of the test (Gallun et al., 2018). Now that it is possible to administer laboratory measures with widely available tablet-based systems, there is no need to compromise methods during clinical translating. The challenge is to select from among the many laboratory measures those that can provide the greatest clinical utility, as defined by efficiency and potential benefit to the patient. This study is part of an ongoing effort to expand the use of current models of auditory dysfunction in clinical assessment by rigorously translating laboratory tests for clinical use.

We followed a systematic approach to the development of a breadth of auditory psychophysical tests while adhering to best practices for the development of clinical assessments. First, we selected a model of auditory processing that is supported by cross-disciplinary hearing science in human, animal, and computational models. In this case, that is the importance of TFS for conveying the instantaneous amplitude of signals, which has a long history in models of monaural and binaural processes (e.g., Licklider, 1948, 1951). Next, we surveyed the existing measures that have been used to demonstrate predictive or diagnostic validity in the context of the model. For TFS, the list of available measures is large and consists of a combination of monaural, diotic, and dichotic tasks spanning several perceptual features including pitch, spatial location, monaural and interaural timing, and tone detection in noise. We narrowed down the list of available TFS measures to those with evidence supporting potential clinical utility and compared them according to criteria of their efficiency and feasibility in a time-constrained clinical battery. The results of this study will be used to guide the translation of a subset of viable clinical measures of TFS processing into a tool for rapid assessment on a platform suitable for clinical deployment, Portable Automated Rapid Testing (PART; Gallun et al., 2015, 2018). PART is currently available as a research tool for the administration of a set of speech and nonspeech measures.

The role of TFS in healthy and impaired speech understanding is supported primarily by the predictive relationships that have been found between impaired performance on psychophysical TFS and various speech tasks. Experiments that limited the amount of TFS available in a speech stimulus have shown that TFS is sufficient (Lorenzi, Gilbert, Carn, Garnier, & Moore, 2006) but not necessary (Van Tasell, Soli, Kirby, & Widin, 1987) for speech intelligibility in quiet, due to the fact that temporal envelope (TE) can be reconstructed from TFS and vice versa (Ghitza, 2001; Zeng et al., 2004). When a background noise is present, there is evidence that TFS is important for the segregation of the target talker from the background (Moore, 2012; Qin & Oxenham, 2003). The ability to take advantage of TFS cues in speech has been shown to be diminished in listeners with impaired TFS processing due to hearing loss (Buss, Hall, & Grose, 2004; Hopkins & Moore, 2010; Hopkins, Moore, & Stone, 2008; Moore, 2008) and aging (Füllgrabe et al., 2015; Lunner, Hietkamp, Andersen, Hopkins, & Moore, 2012). Consistent with these results, impaired TFS processing has been shown to explain individual variability in the ability to understand speech in noise in older listeners with normal hearing (Füllgrabe et al., 2015) and older adults with hearing loss (Hopkins & Moore, 2011; Johannesen, Pérez-González, Kalluri, Blanco, & Lopez-Poveda, 2016; Strelcyk & Dau, 2009).

Below is a list of the measures of monaural and binaural TFS processing selected for the current study, summarized in Table 1. Details about the rationale for the selection of each candidate measure and the published version of the measure to replicate are described in the corresponding Method sections. Measures of monaural TFS processing included a frequency modulation (FM) detection task (FM detection–monaural [FM-M]; Grose & Mamo, 2012), a task requiring the detection of inharmonicity produced by shifting the frequency of all components of a standard harmonic complex by a fixed amount (frequency shift detection [TFS-1]; Hopkins & Moore, 2007), and a gap discrimination (GD) task that involved measurement of the just noticeable increase in interburst interval separating two Gaussian-shaped tone pips (Gallun et al., 2014). Measures of binaural TFS processing included dichotic FM detection (FM/FM; Grose & Mamo, 2012), interaural phase difference (TFS–low frequency [TFS-LF]; Hopkins & Moore, 2011), detection of an interaural time difference (ITD) between Gaussian-shaped tone pips (Gallun et al., 2014), and the detection of an antiphasic tone in diotic noise (N_0_S_π_; D. A. Eddins & Barber, 1998). For completeness, two diotic tasks were included. The first involved FM detection for stimuli identical at the two ears (FM detection–diotic [FM-D]; Grose & Mamo, 2012), and the second was the homophasic tone detection in diotic noise (N_0_S_0_; D. A. Eddins & Barber, 1998).

**Table 1.** Measures of monaural and binaural TFS included in the study.

TFS measure	Stimulus	TFS cue	Study replicated
FM detection–monaural (FM-M)	500-Hz tone	Monaural (right)	Grose & Mamo (2012)
FM detection–diotic (FM-D)		Diotic	
FM detection–dichotic (FM/FM)		Dichotic	
Frequency shift detection (TFS-1)	Complex of missing 100 Hz *f*_0_ centered at 15th harmonic	Monaural (right)	Hopkins & Moore (2007)
Interaural phase difference detection (TFS-LF)	500-Hz tone	Dichotic	Hopkins & Moore (2011)
Interaural timing difference detection (ITD)	750-Hz Gaussian envelope tone pip	Monaural (right)	Gallun et al. (2014)
Gap discrimination (GD)		Dichotic	
Homophasic tone detection in noise (N_0_S_0_)	500-Hz tone, 50-Hz Gaussian noise band	Diotic	Eddins & Barber (1998)
Antiphasic tone detection in noise (N_0_S_π_)		Dichotic	

*Note.* TFS = temporal fine structure; FM = frequency modulation.

The purpose of this investigation was to identify from a set of candidate laboratory measures a subset of measures of TFS processing to include in a practical and cost-effective protocol for possible use in clinic and translational research settings. Based on published evidence of potential clinical utility, a specific version of each task was implemented for comparison. Methods used in each measure were implemented according to their respective publications with few exceptions (noted below). Results were analyzed using the following criteria: (a) agreement with published sources of data for young listeners with normal hearing, (b) relationships among the tasks and measures of speech perception in noise were evaluated using simple linear regression, (c) variance of threshold estimates among subjects with normal hearing, (d) the ability to obtain an accurate threshold estimate in a naïve listener in a clinical setting, and (e) a comparison of the efficiency of each task accounting for differences in methods used in threshold estimation and the variance of threshold estimates obtained. From the above analyses, tasks were selected for inclusion in the battery of tests included in a large-scale, multisite study of listeners with and without suspected auditory processing deficits using PART.

## Method

### Participants

Eleven participants between the ages of 21 and 30 years were recruited for this study. All participants provided informed consent for participation, as approved by the University of South Florida Institutional Review Board. Following consent, all participants underwent a general laboratory intake process that included a brief audiometric evaluation; medical, hearing, and exposure histories; and cognitive screening. Inclusion criteria consisted of normal hearing defined as hearing thresholds of 25 dB HL (ANSI S3.21-2004) or lower at audiometric test frequencies from 250 to 8000 Hz with an interaural asymmetry of less than 10 dB; negative history of head injury, fluctuating hearing, or diagnosed cognitive deficit; and a score of 26 or higher on the Montréal Cognitive Assessment. All participants were paid a nominal hourly rate for their participation.

### Procedure

To facilitate replication, the methods used here were closely matched to those used in the original studies, explained in detail in each section below. Alteration of test parameters was minimized to maintain consistency with those studies to facilitate comparison of thresholds obtained in this study with those reported previously and because of the unknown effect any alteration would have on sensitivity to individual differences in TFS processing. A limited number of methodological changes were made to preserve a consistent test environment throughout the study and to facilitate comparison of within-session variability.

A consistent psychophysical procedure and participant interface was used across all tasks. This was done to avoid changing software, equipment, and participant instructions between tasks and to minimize the need to familiarize subjects with multiple similar procedures. Stimuli were presented in a four-interval, two-cue, two-alternative forced-choice task (2C2AFC), in which the first and fourth intervals contained the standard or “cue” stimulus, whereas the second and third intervals contained either the standard or the target stimulus with equal probability of occurrence in either interval. 2C2AFC was used to provide an exemplar of the standard preceding and following the target interval, which is thought to facilitate comparisons by reducing the need to hold stimuli in short-term memory (Heller & Trahiotis, 1995). The participant interface consisted of a computer monitor displaying four buttons aligned horizontally to represent the four observation intervals. Each button was illuminated during the corresponding interval while stimuli were presented to the participant. Buttons corresponding to the cue intervals (Intervals 10 and 4) were disabled, and the two potential target intervals (Intervals 2 and 3) were enabled while awaiting the participant's selection of a response interval via mouse click. Participants were asked to click on the button that corresponded to the stimulus that was different. Visual confirmation of the correct response interval was provided after each trial.

All tasks were completed in a sound-attenuating booth using the same Sennheiser HD-280 Pro headphones rather than the various headphones used in the original studies. Data collection for each of the tasks was completed in a standard order across three to four test sessions lasting approximately 2 hr each. All tracks included in the final threshold estimate for a given task were performed in a single test session. While the original studies included differing amounts of practice or familiarization, number of adaptive tracks, and stopping rules, the current participants were given a minimum of one adaptive track in each condition as familiarization, and at least three additional tracks were used to estimate threshold. Additional tracks were completed until three tracks were obtained that were consistent according to task-specific criteria of threshold standard deviation and visual inspection of track data. This procedure facilitated the estimation of stability for a given task by comparing thresholds on the initial track to thresholds on the final track after stable performance was achieved. After obtaining three consistent threshold estimates in a given task, thresholds were averaged as a final estimate for each listener. Averaging across reversals and thresholds was performed arithmetically or geometrically according to the cited comparison study.

### Intake Protocol

The intake evaluation included otoscopy, screening tympanometry (Y-226 Hz), and pure-tone thresholds via air and bone conduction. All participants had results within normal limits on each of those indices. In addition to the TFS measures, the study included two measures of speech perception: the Quick Speech-in-Noise Test (QuickSIN; Killion, Niquette, Gudmundsen, Revit, & Banerjee, 2004), a sentence-in-babble test commonly used in clinical settings, and the Spatial Release From Masking for Speech (SR2) task (Gallun et al., 2013; Jakien, Kampel, Stansell, & Gallun, 2017), which is a rapid measure of spatial benefit in a speech-on-speech masking task that has been shown to have good test–retest reliability (Jakien et al., 2017). Speech tasks were used to examine the potential relationship between the TFS tasks chosen and already established and clinically relevant measures of speech understanding in noise in healthy listeners.

### Psychoacoustic Tasks

#### FM Detection

*Rationale*. The detection of FM is an assay of TFS processing because it is thought that listeners use the instantaneous amplitude of the signal to detect small changes in frequency over time by phase locking to peaks in each cycle of the carrier tone. A model of the interference between amplitude and FM was shown to require the addition of monaural TFS cues in order to account for FM thresholds (Ewert, Paraouty, & Lorenzi, 2018). By selecting a low carrier frequency and randomizing the carrier frequency of the unmodulated and modulated stimuli, the potential use of place cues is minimized (Moore & Sek, 1996; Paraouty, Ewert, Wallaert, & Lorenzi, 2016). Cues available through conversion of the FM to amplitude modulation via the auditory filters were avoided by using a low modulation frequency of 2 Hz, where thresholds are well below the limit of 10 Hz at which the amplitude modulation cue can be used (Moore & Sek, 1992, 1994, 1996; Moore & Skrodzka, 2002; Saberi & Hafter, 1995). Impaired performance on FM detection tasks has been attributed to impaired coding of temporal information at the periphery related to aging (Wallaert, Moore, & Lorenzi, 2016) and hearing impairment (Paraouty et al., 2016; Whiteford, Kreft, & Oxenham, 2017). Numerous authors have reported that FM detection accounted for significant individual variability in speech perception in noise in older listeners with sensorineural hearing loss (Buss et al., 2004; Johannesen et al., 2016; Strelcyk & Dau, 2009; Summers, Makashay, Theodoroff, & Leek, 2013), older listeners with normal pure-tone thresholds (Schoof & Rosen, 2014), and listeners with auditory neuropathy (Narne, 2013). To examine the possible relationship between TFS processing and advancing age, Grose and Mamo (2012) measured TFS in groups of young, middle-age, and older listeners with normal hearing using an FM detection task. A subset of the test conditions on which Grose and Mamo observed significant group differences was evaluated here. In the monaural right condition, stimuli were presented to the right ear only. In the diotic condition, identical stimuli were presented to the two ears. In the dichotic FM inverted condition, denoted FM/FM by Grose and Mamo, the signal interval included FM stimuli at both ears; however, the modulator phase was inverted at one ear relative to the other ear.

*Stimuli*. The standard stimulus was an unmodulated tone and, for high FM rates, was perceived to have a fixed pitch height (Zwicker & Fastl, 1990) while the signal stimulus contained FM, which, when detected, was perceived to have a pitch height that fluctuated over time. All stimuli were 1,250 ms in duration, shaped with a 25-ms raised-cosine envelope, and separated by a 200-ms silence between intervals. The standard and cue intervals consisted of an unmodulated pure tone. The target interval contained a pure-tone carrier and a sinusoidal modulator with a frequency of 2 Hz (2.5 cycles of modulation over 1,250 ms). The starting phase of the FM was always 0 radians for the monaural and diotic conditions. The starting phase was 0 radians in one ear and π radians in the other ear in the dichotic condition. To minimize the possibility of using place cues to detect the signal, the carrier frequency was randomly selected on each interval from a uniform distribution ranging from 460 to 540 Hz. The presentation level was 65 dB SPL. The modulator depth was adaptively varied to determine detection threshold using a three-down, one-up stepping rule with equal step sizes down and up. Initially, the depth was scaled by a factor of $\sqrt{2}$. After two reversals, the scaling factor was reduced by $\sqrt{2}$. An additional 10 reversals were completed, and the mean of the last six reversals was taken as the threshold estimate. At least three estimates of threshold were obtained for each condition, and the final threshold was computed as the mean across all three estimates.

**Figure 1.**
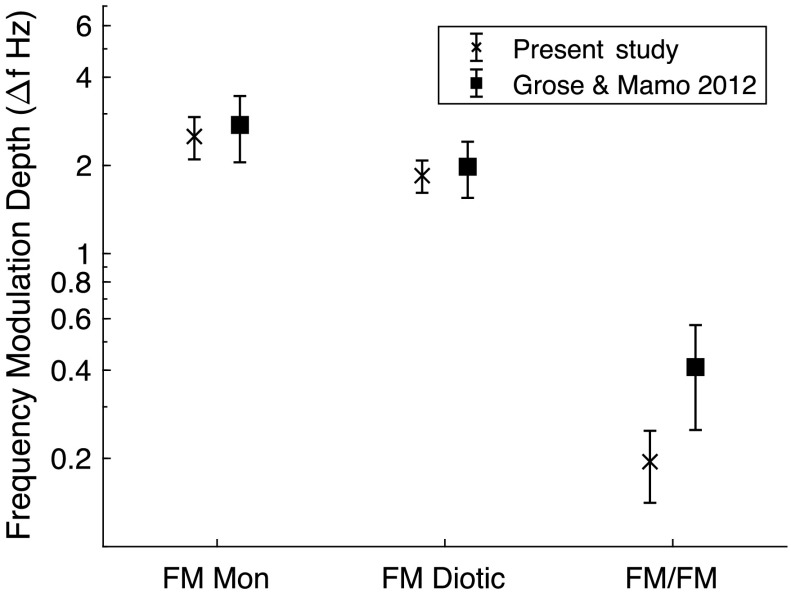
Frequency modulation (FM) detection thresholds as a function of condition for the current data set (x) and the data of Grose and Mamo (2012; squares). Error bars indicate ±1 *SD* from the mean.

*Results*. FM detection thresholds are shown in Figure 1 for three of the different conditions reported by Grose and Mamo (2012), including the monaural condition in the right ear (FM-M; *M* = 2.51 Hz, *SD* = 0.41 Hz), a diotic condition (FM-D; *M* = 1.85 Hz, *SD* = 0.23 Hz), and a dichotic condition in which the signal interval included FM at both ears with the modulation phase inverted at one ear relative to the other (FM/FM; *M* = 0.195 Hz, *SD* = 5.37 × 10^−2^). Similar to the original report, the current data show slightly lower thresholds for the diotic condition than the monaural condition and much lower thresholds for the FM/FM condition. A one-way repeated-measures analysis of variance on the current data set indicated a significant main effect of condition, *F*(2, 9) = 220.07, *p* < .001, η_p_^2^ = .98. Post hoc (Tukey's honestly significant difference) tests indicated that thresholds for the FM/FM condition were significantly lower than either the monaural or diotic conditions. Simple *t* tests were used to compare across data sets; none of which were significant at the *p* = .05 level. Thus, the current FM detection thresholds closely replicated the results reported by Grose and Mamo.

#### Frequency Shift Detection (TFS-1)

*Rationale*. To examine the contribution of auditory temporal processing on reduced speech understanding in aging adults, Hopkins and Moore (2007) created the TFS-1 task that was subsequently modified for clinical use by Moore and Sek (2009). TFS-1 has been shown to relate to speech perception in noise in older adults with sensorineural hearing loss (Innes-Brown, Tsongas, Marozeau, & McKay, 2016; Yeend, Beach, Sharma, & Dillon, 2017) and older adults with normal pure-tone thresholds (Füllgrabe et al., 2015). The TFS-1 task involves discrimination of harmonic complex tones with a fundamental frequency *f*_0_ from similar complex tones with all components shifted by the same number of hertz, producing an inharmonic combination of tones with the same repetition rate but a different waveform morphology. Listeners with normal TFS sensitivity report perceiving this frequency shift as a variation in pitch height. When the harmonic complex contains no resolved harmonics, listeners are thought to use TFS cues to perform the task. However, it may be possible to use place cues from partially resolved harmonics to perform the task (Kale, Micheyl, & Heinz, 2014). Hopkins and Moore (2011) showed that TFS-1 thresholds were not well predicted by frequency resolution as measured using psychophysical tuning curves in subjects with healthy and impaired hearing, suggesting that the ability to resolve differences in place of excitation was not related to performance on the TFS-1 task and supporting the use of TFS cues instead. The methods used here were based primarily on those reported by Moore and Sek.

*Stimuli*. Tone complexes consisted of harmonics of the fundamental frequency*, f*_0_ = 100 Hz*.* The spectrum was shaped in the frequency domain by a Hanning spectral envelope centered on the 15th harmonic, with a nominal passband bordered by the 10th and 19th harmonics and no harmonics included outside that passband. This frequency range is higher than reported previously by Moore and colleagues. In those studies, the flat passband consisted of five harmonics centered at the 11th harmonic, with a 30-dB/octave slope above and below the passband. The reason for using higher harmonics here was to minimize the availability of place pitch cues that are carried by low, resolvable harmonics. By using a complex consisting of higher harmonics, changes due to the signal increment resulted in minimal differences in place on the Greenwood (1990) place–frequency map for a given shift in linear frequency.

The stimuli were presented at 50 dB SPL. Each interval consisted of four consecutive tone bursts 400 ms in duration, shaped by a 20-ms raised-cosine envelope, and separated by a 50-ms interburst interval. Thus, the duration of a single interval was 1,750 ms. Each trial consisted of four such intervals in the 2C2AFC method described above. Harmonic complexes were presented in each of the three standard intervals. In the target interval, the second and fourth bursts were shifted by Δ*F*, producing an inharmonic complex, denoted HIHI by Moore and colleagues, indicating the harmonic and inharmonic sequence of four tone bursts. Starting phases were randomized on each trial to prevent listeners from using the shape of the TE as a consistent cue throughout a track. The adaptive track had a starting frequency shift of 0.5*f*_0_ and followed a two-down, one-up adaptive staircase with equal step sizes up and down. The step size was scaled by 1.25^3^ until the first reversal, by 1.25^2^ until the second reversal, and by 1.25 for the remaining six reversals, for a total of eight reversals. The threshold was the mean of the last six reversals. By rule adopted from Moore and Sek (2009), if the maximum permissible frequency shift was reached twice before the second reversal or once after, the adaptive run would be terminated and 40 trials would be presented at 0.5*f*_0_. This never occurred in practice.

The task was completed in a continuous background of threshold equalizing noise (TEN; Moore, 2004) to render inaudible any distortion products that may have been created by the harmonic complexes used for the task as a result of cochlear nonlinearity or efferent responses. TEN was gated on 1 s prior to the start of the first trial and gated off 1 s after the completion of the final trial in a given adaptive track. For consistency with prior studies, TEN was presented diotically at an RMS level of −15 dB relative to the RMS level of the standard stimulus.

**Figure 2.**
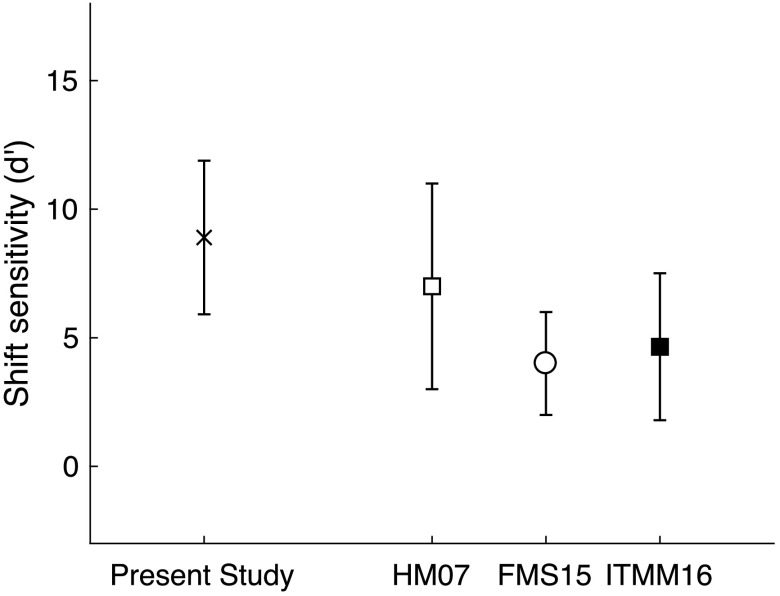
Mean and standard deviation for sensitivity to a shift in frequency for the TFS-1 in studies using similar methods, plotted in a derived estimate of *d*′ described in Hopkins and Moore (2007). Studies differed slightly in the number and shape of harmonic complex stimuli. MH07 is data from Hopkins and Moore for Harmonics 9–13 of 100 Hz in a flat passband and 30 dB/octave slopes above and below. FMS15 is data from Füllgrabe et al. (2015) for a harmonic complex with 30 dB/octave slopes above and below Harmonic 11 of 91 Hz. ITMM16 data were compiled visually from listeners under age of 40 years in Figure 3 of Innes-Brown et al. (2016); stimuli included Harmonics 9–13 of 100 Hz, and filter slope was not reported. Thresholds from this study (frequency shift detection, TFS-1) included Harmonics 10–19 with a Hanning spectral envelope centered at Harmonic 15. Despite minor differences in passband location and shape, sensitivity across the different measures were consistent across studies, with better performance reported in this study discussed in the text.

*Results*. Data for the TFS-1 task are shown in Figure 2, alongside previous studies that used similar methods. Thresholds were obtained as the frequency shift necessary for detection of a change relative to the harmonic standard. Thresholds expressed as the ratio of the change in frequency, Δ*F*, had a mean of 8.42 Hz, a standard deviation of 3.20 Hz, and ranged from 4.74 to 15.40 Hz. To facilitate comparison with previous studies, sensitivity in *d*′ was calculated using the equation 1.63 / threshold * 0.5*f*_0_ (Hopkins & Moore, 2007). This method of calculating sensitivity results in a very high estimate of sensitivity given that 50% detection thresholds for young listeners with normal hearing are well below 0.5*f*_0_ *.* In many studies reporting TFS-1 data, it is necessary to use *d*′ in order to include subjects that were not able to complete the adaptive tracking task; in this study, all subjects completed adaptive tracks successfully. As a result, *d*′ values were even higher than the high *d*′ values reported previously in studies using similar methods. There were two differences in methods in this study compared to previous studies that may have affected thresholds. One difference was the use of 2C2AFC in this study that provided two additional exemplars of the standard stimulus compared to the 2AFC paradigm used in previous studies. This may have resulted in improved detection thresholds or facilitated rapid familiarization with the task. Another difference was the use of a fixed passband that included higher, less resolvable harmonics than previous studies. Young listeners with normal hearing are able to perform the TFS-1 measure using TFS as a cue at harmonics that are not resolvable by the auditory system, but as harmonic number increases, detection threshold also increases as a result of poorer representation of TFS at high frequencies (Hopkins & Moore, 2007; Moore, 2014). The slightly higher harmonics available to listeners in this study compared to previous studies may have resulted in poorer detection thresholds, but this was not the result. It is likely that better TFS-1 thresholds were obtained in this study because all subjects were able to perform the adaptive tracking procedure, and thus, overall performance was exaggerated by the *d*′ calculation.

#### Interaural Phase Difference (TFS-LF)

*Rationale*. An interaural phase difference task, TFS-LF, was introduced by Hopkins and Moore (2010) to evaluate sensitivity to TFS at low frequencies. The task uses a difference in the phase of a tone presented simultaneously to the two ears to evaluate the ability to detect a phase-dependent difference in the instantaneous amplitude of the tones, similar to other interaural phase difference detection tasks (e.g., Lacher-Fougère & Demany, 2005). Using the TFS-LF task, Hopkins and Moore (2011) observed an age-related decline in TFS sensitivity in the absence of both pure-tone threshold elevation and broadened auditory filter bandwidth (Hopkins & Moore, 2011). TFS-LF was a significant predictor of speech understanding in noise in older adults with sensorineural hearing loss (Hopkins & Moore, 2011; Neher, Lunner, Hopkins, & Moore, 2012) and older adults with normal pure-tone thresholds (Füllgrabe et al., 2015).

*Stimuli*. Using the same trial structure as the TFS-1 task, stimuli consisted of four consecutive tone bursts. Each burst had a frequency of 500 Hz and a duration of 400 ms, including 50-ms raised-cosine envelopes. Between each burst was a 20-ms silent period. Between each interval in a trial, there was 200-ms silent period. In the target interval, the first and third tone bursts contained the diotic stimulus and the second and fourth bursts had a different interaural phase. For large differences in interaural phase, the listener perceived the difference as a change in lateralization. Tone complexes were presented at a level of 50 dB SPL. Adaptive tracking was used to determine the minimum detectable difference in interaural phase. The adaptive track started with a phase shift of 180° and followed the tracking rules described above for TFS-1.

**Figure 3.**
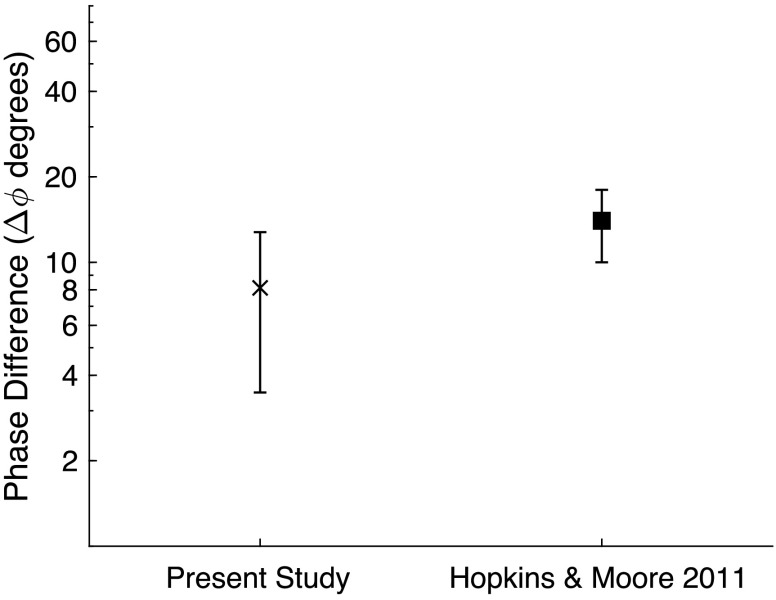
Interaural phase difference threshold (degrees) for this study compared to published data. Mean and ±1 *SD* are plotted as a difference in degrees at threshold.

*Results*. The TFS-LF thresholds in degrees are shown in Figure 3. The mean difference in interaural phase was 9.23°, with a standard deviation of 4.64° and a threshold range from 3.72° to 16.02°. While mean thresholds for the current study are a factor of two smaller than the mean thresholds from the study of Hopkins and Moore (2011), the means in each study were less than 1 *SD* from the other so we did not observe a statistically significant difference in a *t* test at the *p* = .05 level.

#### ITD

*Rationale*. To evaluate temporal processing abilities in older versus younger listeners, Gallun et al. (2014) used three temporal discrimination tasks that included four different stimulus types across the tasks. In this study, two of those tasks were replicated using a stimulus based on the windowed tone-burst stimulus from Gallun et al., referred to here as a “tone pip.” That study concluded that TFS processing resulted in the greatest differences between young listeners and older listeners with hearing impairment, reflecting an independent contribution of age and hearing loss on impaired temporal processing. The brief tone pip stimuli used in the original study had a combination of TE and TFS differences in several conditions. A subset of those conditions were replicated here as the ITD and GD tasks, using parameters selected to enhance the availability of TFS cues. The tone pip in the original study had a carrier frequency of 2000 Hz to match the center frequency of the other three stimuli, which were all based on frequency glides (“chirps”). The auditory system is not well suited to encode carrier TFS cues above roughly 1500 Hz due to substantial reduction in evidence of phase locking to the TFS on the auditory nerve of an animal model (e.g., Palmer & Russell, 1986) and the apparent dominance of redundant place cues for high-frequency carriers (Demany & Semal, 1986; Moore & Sek, 1996). In an attempt to enhance the availability of the TFS cue in the stimulus, the carrier frequency was thus reduced from 2000 to 750 Hz. Brief (4-ms) windows were applied to the carrier (details below), and the resulting tone pips were presented binaurally to assess discrimination of ITDs.

*Stimuli*. Each tone pip had a nominal frequency of 750 Hz, shaped with a Gaussian envelope cropped to *σ* = 6 (±3) within a total duration of 4 ms. Analyses indicated that the spectrum was −50 dB relative to the peak at one octave above and below the nominal frequency. The standard stimulus was presented as a single diotic waveform, giving the listener a perception of the sound being centered in the middle of their head. The target stimulus was delayed in onset and offset at the right ear, producing a binaural ITD in addition to the monaural onset and offset delays and giving the perception of the stimulus being lateralized to the left. Tone pip stimuli were presented at peak-equivalent sound pressure level of 85 dB. Each interval consisted of a single tone pip in each ear, and intervals were padded with 200 ms of silence before and after the pip during which the corresponding button was highlighted. There was an additional 200-ms delay between intervals. A two-down, one-up adaptive tracking rule was used with an initial delay set to 610 μs (0.61 ms). The initial step size was 2^1/2^ and was reduced to 2^1/10^ after the first three reversals. The geometric mean of the interaural delays on trials corresponding to the last six reversals was taken as the threshold for the run. The minimum delay presented was 0.0048 ms, and the maximum delay presented was 34 ms. To accommodate delays less than the sampling period, timing was represented using double-precision floating point values, and the instantaneous amplitude at each sample was computed in each trial.

**Figure 4.**
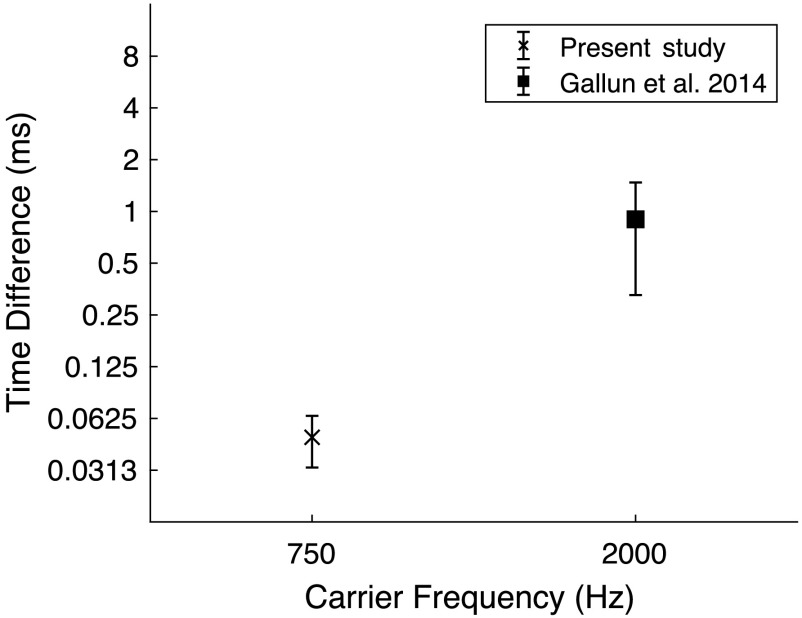
Interaural time difference (ms) for tone pips as a function of tone pip frequency for this study compared to the study of Gallun et al. (2014). Threshold and ±1 *SD* are shown. Note that, in this study, the carrier frequency was lowered to 750 Hz compared to 2000 Hz in the study of Gallun et al. to provide a temporal fine structure (TFS) cue. Substantial improvement in performance in this study suggests that listeners were able to use TFS to perform the task.

*Results*. As shown in Figure 4, the average threshold for the task was 51.2 μs (0.0512 ms), *SD* = 16.1 μs; more than an order of magnitude shorter than the published mean value of 870 μs (0.87 ms) for their group of 37 “younger” listeners (*M*_age_ = 29 years). This difference was significant (*t* = 17.9; *p* < .001, *d* = 7.68) and presumably reflects the difference in carrier frequency between the two studies. The substantial improvement in ITD threshold here relative to the original study is consistent with the notion that, in the original study, TE cues may have been dominant while TFS cues likely were weak at best, while in the current study TFS cues were dominant. Weak TFS cues in the 2000-Hz condition also are consistent with the large error bars, even when plotted on the logarithmic scale of Figure 4. However, the extent to which improved thresholds and reduced variability among subjects with the 750-Hz carrier of the current study resulted from the use of TFS cues or TE cues is unknown.

#### GD

*Rationale*. Tone pips were presented sequentially to one ear to estimate the smallest gap listeners could discriminate from sequential stimuli (Gallun et al., 2014). Gallun et al. (2014) used the same short tone pips for their monaural and binaural tasks and found that the monaural thresholds were very short, relative to those observed for longer duration stimuli of similar bandwidth (e.g., Ozmeral, Eddins, & Eddins, 2016). In addition, a wide-band “chirp” stimulus was examined in order to determine whether the task was purely TE or TE and TFS. When the phases of the frequency components were randomized, thresholds were 50% greater than when the chirps were rising or falling in frequency. This suggests that TFS is important for this task and may explain the shorter gaps at which the task could be performed relative to the long-duration stimuli. Tone pips were presented sequentially to one ear to estimate the smallest gap listeners could discriminate from sequential stimuli (Gallun et al., 2014). Temporal gap stimuli have been used to show differences in temporal processing associated with aging (e.g., Ozmeral et al., 2016), hearing loss (e.g., Grose, Eddins, & Hall, 1989), and developmental auditory processing disorders (Irwin, Ball, Kay, Stillman, & Rosser, 1985; Keith, 2000; but also see Hall & Grose, 1994; Levi & Werner, 1996). The task was to discriminate two sequential tone pips with no “interpip” delay from two sequential tone pips with a delay between them referred to as a silent gap. Although technically a gap detection task, the use of Gaussian envelope tone pips allowed the listener to detect two discrete pips even when no gap was present between them, and so Gallun et al. referred to this as *gap discrimination*. As with tone pip ITD, both TE and TFS cues were present in the stimuli for use in discrimination of the gap duration.

*Stimuli*. The tone pip stimuli were identical to those described for the ITD task, presented sequentially to a single ear rather than to two ears. Tone pips were presented at a peak-equivalent level of 85 dB SPL. The standard stimulus consisted of two sequential tone pips with no delay. The target stimulus introduced a brief delay or temporal gap between the first and second stimulus. The delay or gap duration was initially set to 4 ms. The duration of the gap was adaptively varied using a two-down, one-up adjustment rule and a logarithmic step size. The initial step size was a multiplicative factor of 2^1/2^ and was reduced to 2^1/10^ after the first three reversals. The geometric mean of the last six reversals was taken as the threshold estimate.

**Figure 5.**
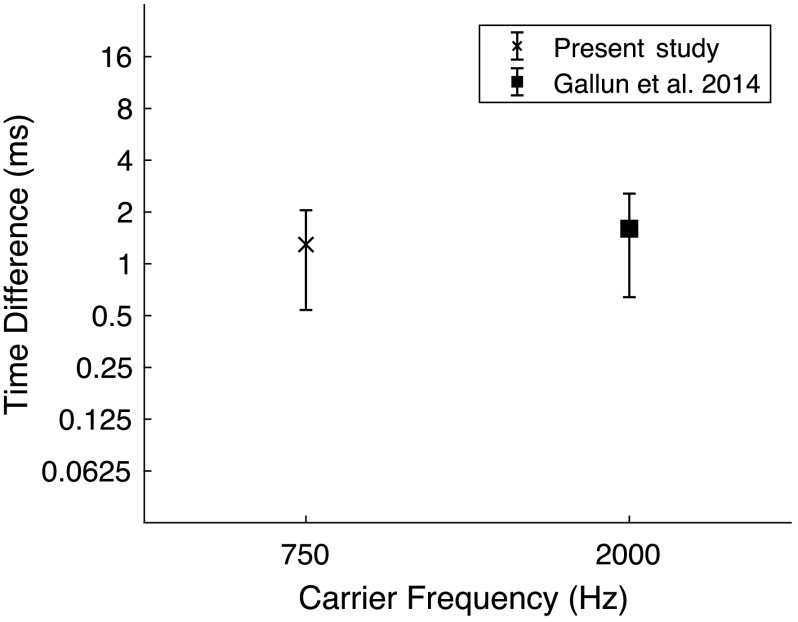
Gap discrimination (ms) for tone pips as a function of study. The relevant data from Gallun et al. (2014) are shown for comparison with this study. Note that carrier frequency was changed to 750 Hz in this study from 2000 Hz to provide a temporal fine structure cue. No change in performance was observed, suggesting listeners used the same temporal envelope cue to perform the task.

*Results*. The average GD thresholds of the current study (see Figure 5, left) are very similar to those reported in the original study (Gallun et al., 2014) despite the marked difference in carrier frequencies (i.e., 750 vs. 2000 Hz). The mean threshold was 1.45 ms (*SD* = 0.755 ms), and thresholds ranged from 0.604 to 2.89 ms. In this case (unlike the ITD case above), the similarity in thresholds is inconsistent with a dominant TFS cue, since such a cue would be more poorly coded at 2000 Hz than at 750 Hz. Rather, the similarity is consistent with the use of a TE cue. As noted in the original report, the thresholds obtained are shorter (approximately 1.5 ms) than other reports with tonal stimuli (approximately 5 ms; e.g., Shailer & Moore, 1987) with long-duration stimuli but are consistent with those reported by Schneider, Pichora-Fuller, Kowalchuk, and Lamb (1994), who also used very short gap markers. Such a threshold duration is comparable to one period of a 750-Hz tone pip and three periods of a 2000-Hz tone pip.

#### Tone-in-Noise Detection (N_0_S_0_, N_0_S_π_)

*Rationale*. The tone-in-noise paradigm has a long history as an index of binaural temporal processing with the notion that the addition of an interaural phase difference to the signal in the N_0_S_π_ condition creates a large change in interaural normalized cross-correlation relative to the standard intervals, and this facilitates detection over the N_0_S_0_ condition (e.g., Carney, Heinz, Evilsizer, Gilkey, & Colburn, 2002; van der Heijden & Trahiotis, 1997), which in turn relies on a nonlinear combination of energy, TFS and TE cues for detection (Mao, Vosoughi, & Carney, 2013). The difference between N_0_S_0_ and N_0_S_π_ detection thresholds or the binaural masking level difference (BMLD) is thus an index of sensitivity to differences in interaural timing coded, at least in part, by TFS. Several authors have shown a significant relationship between tone-in-noise detection and speech understanding in noise (Papakonstantinou, Strelcyk, & Dau, 2011; Roup & Leigh, 2015; Strelcyk & Dau, 2009). The use of a narrowband noise masker generally results in a much larger BMLD than a broadband noise masker (Hall & Harvey, 1985), potentially increasing the ability to separate differences among listener populations. The methods used here were adapted from the study by A. C. Eddins and Eddins (2018).

*Stimuli*. Masked signal detection was measured under homophasic (N_0_S_0_) and antiphasic (N_0_S_π_) conditions. The masker stimulus was presented continuously in the background throughout the test. The masker consisted of a 50-Hz narrowband noise centered at 500 Hz. The noise stimulus was generated in the frequency domain by selecting magnitude from a Rayleigh distribution and phase from a uniform distribution 0 to 2π radians and performing an inverse Fourier transform (Hartmann, 2004). Stimulus generation was as described in A. C. Eddins and Eddins (2018). The tonal signals had a frequency of 500 Hz and duration of 400 ms with 20-ms rise–fall windows. The standard and cue intervals consisted of noise only with a duration of 400 ms. Intervals were indicated by sequential highlighting of response buttons, with 200-ms separation between intervals. Noise was presented at a fixed level of 77 dB SPL, and tone level was adaptively varied to find the lowest signal-to-noise ratio (SNR) at which the listener could detect the tone in a three-down, one-up tracking paradigm. The starting SNR was 10 dB in the homophasic condition and −2 dB in the antiphasic condition. An equal step size of 2 dB was used up and down for a total of 60 trials. Threshold was taken as the mean of the last six reversals.

**Figure 6.**
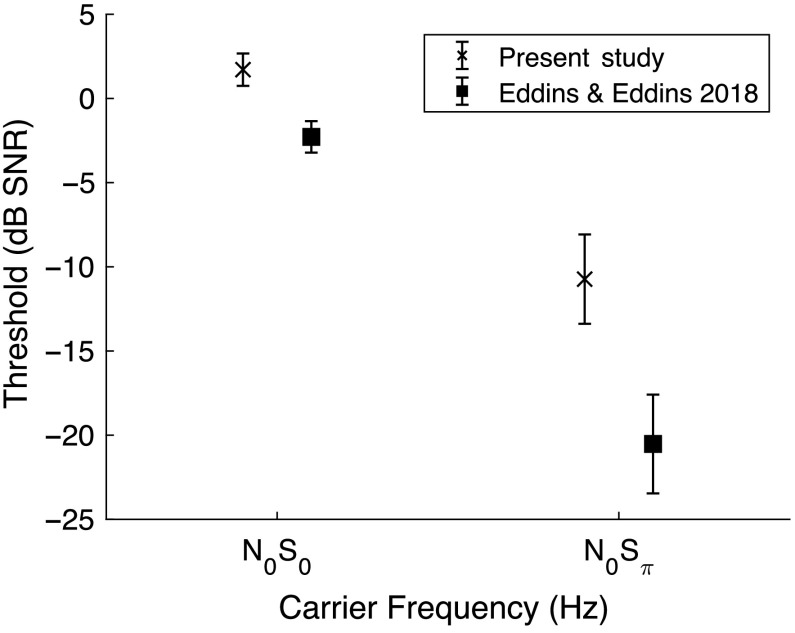
Threshold as a function of stimulus condition for the binaural masking level difference tasks. Thresholds are specified in dB SNR and mean and ±1 *SD* are shown. Differences in threshold across studies were observed, but thresholds for both studies were in the range observed in studies that used slightly different methods.

*Results*. As shown in Figure 6, mean thresholds for the N_0_S_0_ condition were 1.71 dB SNR (*SD* = 0.96 dB) and ranged from 0.11 to 3.00 dB SNR. In the N_0_S_π_ condition, mean thresholds were −10.73 dB SNR (*SD* = 2.65 dB) and ranged from −14.22 to −6.44 dB SNR. The BMLD values for the current study (*M* = 12.44 dB SNR, *SD* = 2.95 dB) were smaller than the 18.24 dB average reported by A. C. Eddins and Eddins (2018; *t* = 4.93, *p* < .001, *d* = 2.17). N_0_S_0_ thresholds were higher in the current study, which would favor larger rather than smaller BMLDs (*t* = 9.61, *p* < .001, *d* = 4.20). The N_0_S_π_ thresholds, on the other hand, were considerably higher in the current study than reported by A. C. Eddins and Eddins (*t* = 8.03, *p* < .001, *d* = 3.51), revealing the primary source of smaller BMLDs. Minor procedural differences between the two studies are not consistent with such large threshold differences but are worth of description here. In the A. C. Eddins and Eddins study, a long-duration noise was mixed with the tonal signal using an analog mixer (Tucker-Davis Technologies SM3). The S_0_ and S_π_ signals were differentiated by means of a phase inverter on the SM3 mixer, and the signal-plus-noise stimuli were delivered via Etymotic ER-2 insert earphones. In this study, the signal phase was controlled digitally, the noise and signal were mixed digitally, and the stimuli were presented via Sennheiser HD-280 Pro circumaural headphones. None of these differences appear to account for the threshold differences observed.

Several investigators have reported somewhat larger variability in BMLD thresholds across subjects when using narrowband noise maskers (e.g., D. A. Eddins & Barber, 1998; Hall & Grose, 1995; Jain, Gallagher, Koehnke, & Colburn, 1991). Interestingly, the BMLDs reported by D. A. Eddins and Barber (1998), for a 500-Hz signal frequency and 50-Hz band, were only slightly larger than the BMLD reported here (15 vs. 12 dB). The variability across subjects in that study, however, was so great that they separated listeners into two groups—one with “large” BMLDs (approximately 18 dB) and one with “small” BMLDs (approximately 10 dB). BMLDs in the current study are closer to the small BMLD group of D. A. Eddins and Barber, whereas BMLDs in the A. C. Eddins and Eddins (2018) study are closer to the larger BMLD group of D. A. Eddins and Barber's study. In designing their study, A. C. Eddins and Eddins considered that the variability in D. A. Eddins and Barber's study might be due to the use of simultaneous gating of the signal and noise, resulting in a burst condition known to impact signal detection values (e.g., Fantini, Moore, & Schooneveldt, 1993; McFadden & Wright, 1992). The lack of variability in that study supported such a possibility. The variability reported here, however, brings into question that source of variance. Perhaps population sampling is the most likely source of variance, but that cannot be ruled in or out with certainty. It is notable that the BMLDs reported by A. C. Eddins and Eddins varied little across their 12 young, normal hearing listeners, and the standard deviation reported previously was similar to that reported here (2.37 dB, *n* = 10 and 2.95 dB, *n* = 11, respectively). Finally, a potential limitation of BMLD tasks with low signal frequencies (e.g., 500 Hz) and narrow noise bandwidths (e.g., 50 Hz) is that both TE and TFS cues are introduced in the signal interval. Indeed, Bernstein and Trahiotis (1992) showed that, even when TFS cues were rendered unavailable by testing at a center frequency of 4000 Hz, a robust BMLD could be achieved on the basis of interaural temporal disparities carried in the TE. The low-frequency, narrowband noise maskers (as used here) contain fluctuations over a continuous range of time scales spanning TE and TFS coding. Thus, differences among listeners and among studies may simply reflect differences in TE versus TFS cue weighting, as suggested by A. C. Eddins and Eddins to account for differences in BMLD values across age. Unless such weighting can be quantified, however, BMLD may not be well suited as an index of binaural TFS coding.

#### Speech in Noise

*Rationale*. Two common measures of speech intelligibility in the presence of background competition are the QuickSIN (Killion et al., 2004) and SR2 (Gallun et al., 2013). For both QuickSIN and SR2, TFS cues present in the target and background speech signals may be used by the listener. SR2 provides cues to target and masker location via ITD cues carried by both TFS and TE that listeners use to segregate the talkers by location (Ellinger, Jakien, & Gallun, 2017). The extent to which TFS specifically contributes to performance in the QuickSIN or SR2 or to what extent variation in performance on TFS measures in young, asymptomatic listeners account for variation in QuickSIN or SR2 performance is unknown. These two tests were completed to evaluate whether or not there was a strong relationship between TFS measures and speech in noise.

*Stimuli*. The QuickSIN consists of sentences spoken by a female talker presented in a background of competing talkers. QuickSIN was performed monaurally in both ears using the audio CD presented via Etymotic ER-3A insert phones. The SR2 consists of formulaic sentences spoken simultaneously by three male talkers, one target talker identified by a key word and two background talkers, and is based on the coordinate response measure corpus (Bolia, Nelson, Ericson, & Simpson, 2000). SR2 was used to evaluate the role of interaural timing cues to talker location in spatial release from masking for speech. The listener must identify the target talker by the key word and select a color–number combination from a response grid of four colors and nine numbers. Dichotic stimuli were generated by convolving the stimuli with generic head-related impulse responses and presented binaurally via HD-280 Pro headphones. The use of generic head-related impulse responses means that spectral cues to location were obscured, but interaural timing cues were preserved in both the TFS and the TE. The presentation level was 65 dB SPL RMS. The target talker was always presented in front (0° azimuth), and background talkers were either presented collocated in front or spatially separated (±45° azimuth). Two progressive tracks were presented in collocated and spatially separated conditions in descending target-to-masker ration (TMR), and the TMR corresponding to 50% correct was estimated from the average number of color–number targets correctly identified in each condition. Prior to testing, the listener was familiarized with the task by listening to one exemplar at each TMR in each condition.

*Results*. All listeners performed QuickSIN within the normal range in both ears. The mean thresholds were 0.27 dB SNR loss in the left (*SD* = 1.17 dB, range: −2 to 2 dB) and 0.54 dB SNR loss (re: SNR average for normative data) in the right (*SD* = 0.99 dB, range: −2 to 1 dB). SR2 performance was consistent with published data for young listeners with normal hearing (Jakien et al., 2017). In the collocated condition, the TMR necessary for 50% correct identification was 1.14 dB (*SD* = 0.78 dB, range: 0–2.5 dB). Listeners were able to benefit from a spatial separation of the background talkers, with an improvement of the TMR at 50% correct of −6.23 dB (*SD* = 1.98 dB, range: −8.5 to −1.0 dB). A single listener was an outlier in the spatially separated condition, with a −1 dB TMR corresponding to a *z* score of 2.44 based on normative data (Jakien & Gallun, 2018). With that listener excluded, the group mean was −6.75 dB TMR (*SD* = 1.01 dB, range: −8.5 to −5.0 dB), and all were within ±1 *SD* of predicted values for collocated, separated, and masking release based on age and pure-tone average (Jakien & Gallun, 2018). It is unclear why a single listener apparently received minimal spatial release from masking; their performance was not exceptional on any other test.

## Discussion

### 

#### Relationships Among Tasks

A bivariate correlation analysis and a regression analysis, with each measure as the dependent variable and each other measure as the predictor variable, were performed as a shotgun approach to examine the relationships among each of the TFS measures. To account for differences in range and scale among the various measures, all thresholds were normalized prior to evaluating these comparisons by taking the log of thresholds represented in linear units (TFS-1, TFS-LF, GD, and ITD) and converting all thresholds to *z* scores using the sample mean and standard deviation. Bivariate correlations revealed a single relationship among the data accounting for a significant amount of the variance without correcting for multiple comparisons, between the binaural tasks of FM/FM dichotic and N_0_S_π_ (*r* ^2^ = .38, *p =* .043), both of which rely on the use of a difference in instantaneous phase of a 500-Hz tone between the ears. Regression analysis revealed no significant relationships among the QuickSIN, the SR2, and the TFS tasks. We interpreted the lack of relationships among the tests as consistent with the assumption that variance in our data was due to random noise resulting from normal variation in the healthy population and measurement error. All participants were young, had thresholds on all tests consistent with published data for normal hearing listeners, and were within the normal range on speech-in-noise tests. Consistent with this interpretation, we used intersubject variance in analyses below as an indication of the magnitude of an effect that would be required to detect impaired performance. However, due to the number of participants in the study, it did not have sufficient power to rule out systematic relationships among the data. We expect to see a strong predictive relationship between TFS and speech understanding in noise in populations with impaired temporal coding, as demonstrated in many of the studies replicated here.

#### Measures Suitable for Clinical Use

To determine which measures would be most suitable for an efficient, rapid clinical protocol, the results were compared in three ways—listener response consistency, variance of the threshold estimate, and test duration.

**Figure 7.**
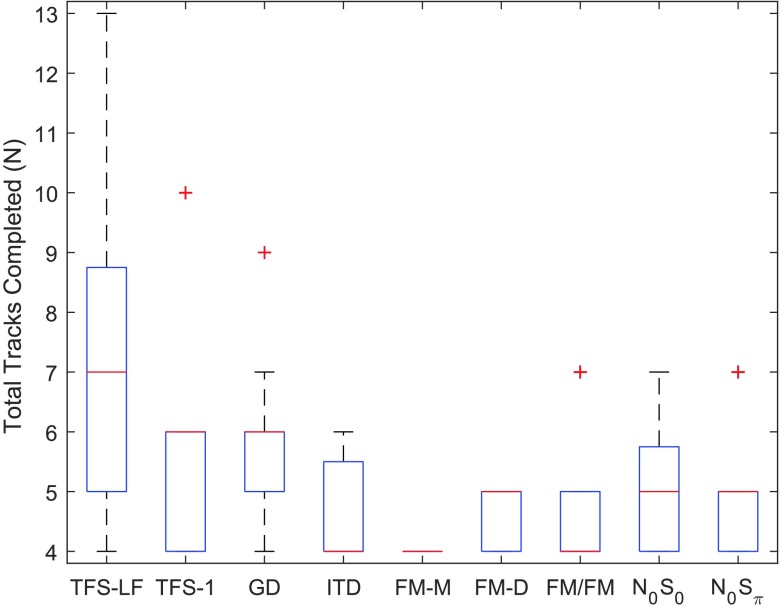
Total number of tracks completed in each measure, including an initial practice track not included in the final threshold calculation and at least three test tracks. Additional tracks were added as needed to obtain a reliable threshold estimate. TFS-LF = TFS–low frequency (interaural phase difference detection); TFS-1 = frequency shift detection; GD = gap discrimination; ITD = interaural time difference; FM-M = FM detection–monaural; FM-D = FM detection–diotic; FM/FM = FM detection–dichotic; N0S0 = homophasic tone detection in noise; N0Sπ = antiphasic tone detection in noise.

*Response consistency*. Listener response consistency was quantified by counting the number of tracks necessary to obtain a consistent threshold for a given listener. Participants completed adaptive tracks until three consistent threshold estimates (track converged and threshold estimate with standard deviations from the mean) were obtained. A minimum of four tracks were completed for each task, including an initial practice track. Figure 7 displays the number of tracks required per task by each individual listener to achieve such consistency. Among the least consistent tasks were TFS-LF, TFS-1, and GD, with six or more tracks required to achieve a valid threshold estimate. The three FM tasks were among the most consistent, as most listeners required no more than one additional track to obtain a consistent listener performance.

**Figure 8.**
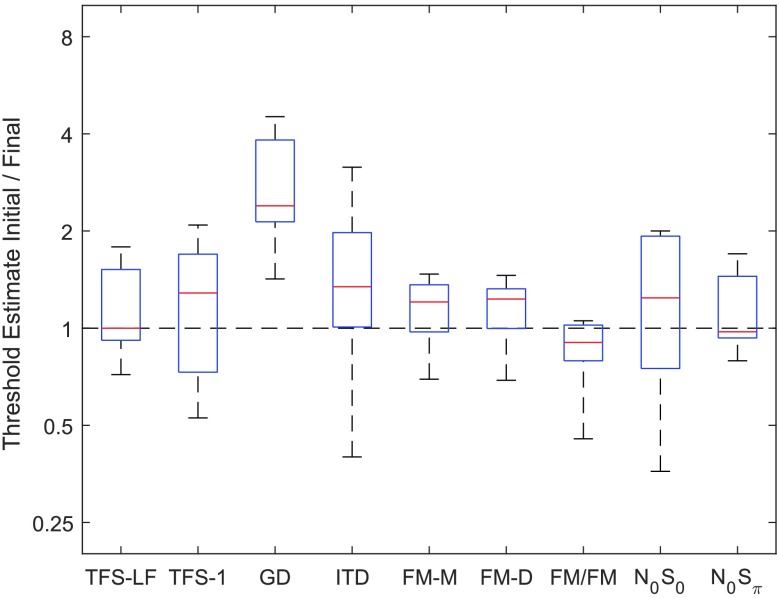
Comparison of the threshold obtained in the initial track to the final threshold estimate for each measure. The dashed line at unity represents equal thresholds for initial and final estimates. TFS-LF = TFS–low frequency (interaural phase difference detection); TFS-1 = frequency shift detection; GD = gap discrimination; ITD = interaural time difference; FM-M = FM detection–monaural; FM-D = FM detection–diotic; FM/FM = FM detection–dichotic; N0S0 = homophasic tone detection in noise; N0Sπ = antiphasic tone detection in noise.

*Clinical efficiency*. For a clinically efficient protocol, it is ideal to minimize the time required to complete the given task. Thresholds obtained in the initial track completed by a naïve subject are expected to provide an index of performance equivalent to the asymptotic threshold obtained following a rigorous laboratory procedure, ideally with minimal difference in the variance of the estimate. To evaluate systematic differences in thresholds over repeat tracks, an index of stability was computed by dividing the threshold obtained in the first track by the threshold obtained for the final track. Greater change from the initial to the final estimate of threshold indicates that some aspects of listener performance (i.e., criterion, strategy, familiarity) change rapidly within a single test session. This comparison is displayed in Figure 8 for each task. The FM detection tasks are among the most stable, with a ratio of less than 0.5 between the initial and final tracks. GD had the least stable thresholds, with an initial threshold two to four times greater than the final threshold estimate for most listeners.

**Figure 9.**
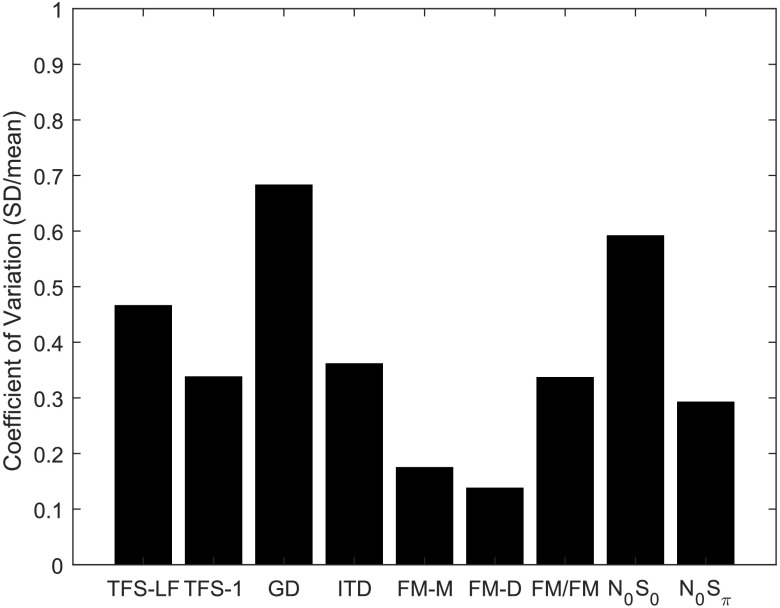
Coefficient of variation (standard deviation divided by mean) for each measure. TFS-LF = TFS–low frequency (interaural phase difference detection); TFS-1 = frequency shift detection; GD = gap discrimination; ITD = interaural time difference; FM-M = FM detection–monaural; FM-D = FM detection–diotic; FM/FM = FM detection–dichotic; N0S0 = homophasic tone detection in noise; N0Sπ = antiphasic tone detection in noise.

*Overall efficiency*. To compare test efficiency across tests, normalized variance was quantified in two ways. The first metric of variance was based on the distribution of scores across listeners. Since there was no a priori reason to expect a group of young listeners with normal hearing to vary in their ability to perform TFS tasks, we can assume that variance within this group is representative of the population variance for each test. The test with the lowest population variance should be most sensitive to TFS impairment given an equal relative mean difference between the healthy group and the group with impairment. Figure 9 shows the coefficient of variation for each of the TFS tests, calculated as the standard deviation divided by the absolute value of the sample mean. GD and N_0_S_0_ were the most variable across listeners, with a standard deviation greater than half the sample mean. Monaural and diotic FM were the least variable—a result indicating that these tests would be most sensitive to small differences between the healthy group and the group with impairment.

**Figure 10.**
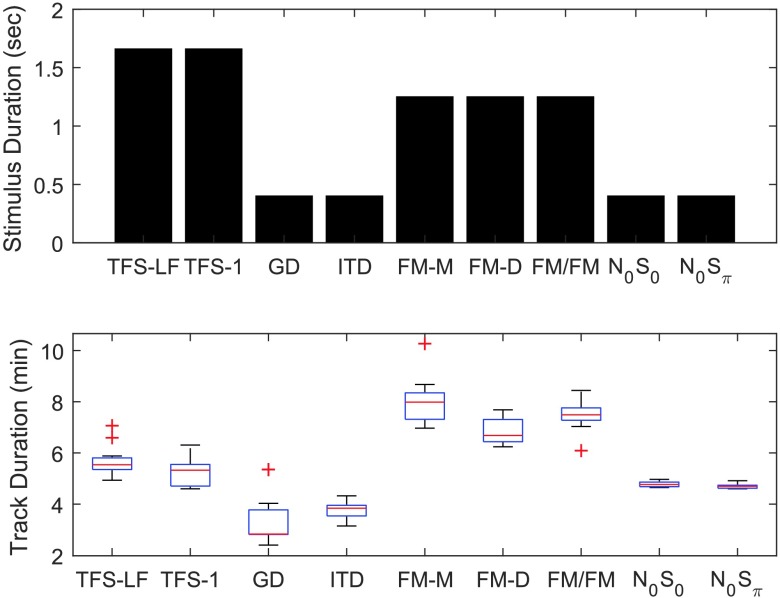
Stimulus duration in seconds (top panel) and track duration in minutes (bottom panel) for each measure. TFS-LF = TFS–low frequency (interaural phase difference detection); TFS-1 = frequency shift detection; GD = gap discrimination; ITD = interaural time difference; FM-M = FM detection–monaural; FM-D = FM detection–diotic; FM/FM = FM detection–dichotic; N0S0 = homophasic tone detection in noise; N0Sπ = antiphasic tone detection in noise.

Clinical efficiency must be determined by comparing the utility of performing the test to the cost of performing the test. Utility remains to be establish in further stages of this ongoing research following the translation and validation of efficient versions of the measures selected in the present experiment, but we shall assume that there is approximately equivalent utility of TFS testing across all measures or within the monaural and dichotic domains. Assuming equivalent utility, the present data allow for a comparison of efficiency by comparing cost among the candidate tasks. The differential cost of performing each of the candidate tasks is determined primarily by the time necessary to perform the task. The time of each TFS test was quantified based on the duration of a given stimulus presentation, the duration of a track, and the total duration needed to obtain a reliable threshold estimate. TFS tests were administered using a consistent 2C2AFC paradigm, requiring the presentation of the basic stimulus or stimulus sequence in a total of four intervals per trial. A clinical version of a given test may present as few as one interval, or sequence the intervals in a continuous task, so a comparison of interval duration facilitates comparison across the tests independent of procedure. Figure 10 shows the duration of a single interval (top panel), and the median duration of a track across listeners (bottom panel). The TFS-LF and TFS-1 tasks had the longest stimulus duration, resulting in relatively long tracks. The FM tests were time consuming relative to the tone pip gap and ITD tests. N_0_S_0_ and N_0_S_π_ had a short stimulus duration, and track duration was consistent due to the use of a fixed number of trails. The number of trials in a given track is directly proportional to the variance of the threshold estimate, but the relationship between stimulus duration and threshold is measure dependent.

**Figure 11.**
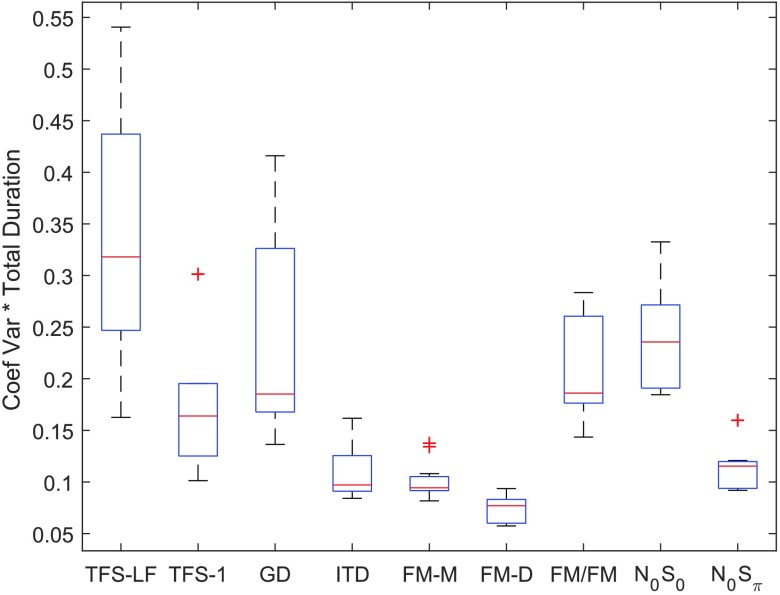
The product of coefficient of variation and total test duration for each measure as an index of the efficiency similar to sweat factor. Higher numbers indicate that more time was needed to obtain the same accuracy of threshold estimate. TFS-LF = TFS–low frequency (interaural phase difference detection); TFS-1 = frequency shift detection; GD = gap discrimination; ITD = interaural time difference; FM-M = FM detection–monaural; FM-D = FM detection–diotic; FM/FM = FM detection–dichotic; N0S0 = homophasic tone detection in noise; N0Sπ = antiphasic tone detection in noise.

A time-adjusted variability index was computed, as shown in Figure 11. The index is a more accurate estimate of the efficiency of the TFS measures than the variance of the threshold estimate alone because it takes into account the fact that the variance is proportional to the number of trials used to obtain the estimate. Time-adjusted variability was computed by multiplying the coefficient of variance by the total test duration in hours. The index is akin to sweat factor (Taylor & Creelman, 1967), except that total duration was used rather than track duration to incorporate differences in implementation across tests. According to this index, the monaural and diotic FM tests were the most efficient, followed by N_0_S_π_ and ITD. Although fast, the GD task was inefficient, as it suffered from high variability across listeners. One goal of this study was to select, among competing tests of TFS processing, a subset of tests suitable for clinical implementation. Based on this index, FM-M was a clear winner among tests of monaural TFS. FM-D was the most efficient diotic test according to this statistic, and ITD and N_0_S_π_ were the most efficient dichotic tests. These data provide support for the suggestion made by various authors that N_0_S_π_ should be used rather than the complete BMLD, since N_0_S_0_ doubles the test time and is highly variable within young listeners with normal hearing. None of the measures used in this study were subjected to an optimization for procedural efficiency, and it is possible that this index of efficiency could be improved. However, there is no reason to suspect that procedural optimization applied to all measures would change the relative efficiency across measures.

*Limitations*. Decisions in the selection of stimulus and procedural parameters influenced the estimates of efficiency in this study. Choices such as stimulus duration, 2AFC versus 2C2AFC procedures, and adaptive tracking rules, if changed, would lead to different final estimates of coefficient of variation and sweat factor. In the case of TFS-1 and TFS-LF, the original studies used a long-duration interval consisting of a pattern of alternating stimuli replicated here, but they used a two-alternative procedure resulting in a lower trial duration than was used in this study. Any of a number of procedural changes could bias the result to favor a given test. However, due to the large differences in estimated efficiency across tasks, we believe that the conclusions of this study would remain valid following optimization of each task. Furthermore, it was our intent to replicate procedures used in seminal studies that reported deficits in TFS processing of potential clinical utility as discussed above.

It is possible for an optimized version of monaural and dichotic TFS measures to be implemented in future arms of the study, given suitable validation that the changes did not degrade the clinical utility. One strategy would be to shorten the duration of the stimuli used. The stimulus parameters used here were determined in the original publications cited, and those studies did not have the same focus on clinical practicality. The test protocol also may be optimized by decreasing the number of minimum tracks required. In the clinical setting, it will not be necessary to complete long tracks for the tasks and judge that there is sufficient listener consistency and threshold stability. Due to the high consistency of the measures evaluated here between the initial and final thresholds estimates, a single, brief track will likely be sufficient in both monaural and dichotic TFS measures.

It is the intent of the investigators to continue evaluating these and other tasks with a larger sample size and to evaluate group comparisons including older listeners, listeners with hearing impairment, and listeners with traumatic brain injury. Comparisons of both the QuickSIN and the SR2 task should be continued with a larger sample size. As no correlation was found between the QuickSIN and SR2, each should be evaluated as it is thought it may be measuring different deficits of speech understanding in noise unless it becomes clear with clinical populations that such correlations (and presumably underlying abilities) arise. The larger test protocol noted above will include optimized versions of the TFS and other tasks implemented on tablet computers with consumer headphones in order to approach an efficient platform (PART) for clinical use. Data collected using this consumer-based platform then will be compared to data collected with laboratory systems and methods.

#### Clinical Significance

Combined across the numerous TFS measures that have been evaluated, there is substantial evidence that TFS assessment may be useful in the diagnosis of hearing disorders—in improving our understanding of complex patterns of disorder that may arise from various insults to the peripheral or central auditory system. There is potential for TFS to be incorporated into treatment, for example, in predicting benefit of amplification based on the ability to code temporal information in signals that are audible or in determining limits to the acceptable distortion introduced by hearing aid signal processing algorithms such as noise reduction. Despite a long history of rigorous research, including animal and computational models, and mounting evidence for the clinical utility of TFS, it is typically not included in batteries of tasks intended to characterize auditory processing ability. This may be due to the ongoing debate about the peripheral coding of TFS and the common use of multiple definitions of TFS, which has resulted in many different measures that ostensibly test the same ability. Although no specific clinical recommendation can be made on the basis of this study, this work serves as an intermediate step in the application of basic scientific work on the perception of TFS by healthy listeners and listeners with impairment and the use of TFS in the clinic. By replicating and comparing the TFS measures that have demonstrated potential clinical utility, the results of this study can be used to guide the selection of TFS tests for use in future studies and facilitate the use of TFS in battery assessments like PART. It is our hope that this will contribute to improving our understanding of TFS in healthy listeners and listeners with impairment and the characterization and treatment of disorders that affect performance on TFS tests.

## Summary and Conclusions

The goal of the current study was to evaluate a set of measures that are candidates for inclusion in an efficient clinical protocol that includes the evaluation of TFS processing among a number of other auditory perceptual abilities. The findings of the study are summarized in Table 2.

**Table 2.** Summary of the thresholds and comparison metrics obtained in this study.

Measure	Threshold estimate	Tracks	Initial estimate/final	Stimulus duration	Task duration	Coefficient variation	Sweat factor
	*M, SD*	*Mdn*	Median ratio	Median min.	Median min.	*SD*/*M*	Median Hr.
TFS-LF	9.23°, 4.64°	7	1.0	5.5	41.0	0.466	0.318
TFS-1	8.42 Hz, 3.20 Hz	6	1.3	5.3	29.1	0.338	0.164
GD	1.45 ms, 0.775 ms	6	2.4	2.8	16.3	0.683	0.185
ITD	51.2 μs, 16.1 μs	4	1.5	3.8	16.1	0.361	0.097
FM-M	2.51 Hz, 0.41 Hz	4	1.2	8.0	32.4	0.175	0.094
FM-D	1.85 Hz, 0.23 Hz	5	1.1	6.7	33.5	0.138	0.077
FM/FM	0.195 Hz, 0.054 Hz	4	0.9	7.5	33.2	0.337	0.186
N_0_S_0_	1.71 dB SNR, 0.96 dB SNR	5	3.3	4.8	23.9	0.592	0.236
N_0_S_π_	−10.73 dB SNR, 2.65 dB SNR	5	1.1	4.7	23.7	0.292	0.115

*Note.* Median min. = median duration in minutes; Median Hr. = median duration in hours; TFS-LF = TFS–low frequency (interaural phase difference detection); TFS-1 = frequency shift detection; GD = gap discrimination; ITD = interaural time difference; FM-M = FM detection–monaural; FM-D = FM detection–diotic; FM/FM = FM detection–dichotic; N_0_S_0_ = homophasic tone detection in noise; N_0_S_π_ = antiphasic tone detection in noise.

The primary conclusions of this study are that FM-M is the best among the indices of monaural TFS and that several dichotic tasks remain excellent candidate indices of binaural TFS. ITD with Gaussian envelope tone pips was the most efficient measure among the dichotic tasks but showed a familiarization effect in the change in threshold from initial to final estimate that would not be ideal for a rapid test protocol. N_0_S_π_ was the next most efficient dichotic measure after ITD, and listeners improved less than 10% from the initial to the final threshold estimate. FM/FM was less efficient than ITD and N_0_S_π_, but performance was as good on the initial track as in the final estimate. FM/FM has greater construct validity than the other dichotic TFS measures because of its widespread use in studies evaluating the role of place and timing cues (summarized above) and associated modeling (Ewert et al., 2018), but large differences across studies with very similar methods indicate that there is more to learn about individual differences in performance on FM/FM. Both ITD and N_0_S_π_ have unresolved controversy regarding the use of TFS cues to perform the task, as discussed above. FM-D was found to be very efficient and suitable for a rapid protocol, but ambiguity regarding the role of the binaural system in a diotic task means that it is not preferable to the various dichotic tasks that were slightly less efficient. Going forward, we intend to use FM-M to assess monaural TFS and to evaluate ITD, N_0_S_π_, and FM/FM in older listeners with and without hearing loss. We will ultimately use diagnostic power to determine which measure provides the best index of binaural temporal resolution.
